# The efficacy and safety of auricular acupoint therapy on treating functional dyspepsia with insomnia: study protocol for a randomized controlled trial

**DOI:** 10.3389/fmed.2025.1496502

**Published:** 2025-03-12

**Authors:** Meng-Yuan Shen, Qin-Yi Lou, Shan Liu, Ze-Jiong Li, Tian-Chen Lin, Rong Zhou, Dan-Dan Feng, Dong-Dong Yang, Jian-Nong Wu

**Affiliations:** ^1^The First Affiliated Hospital of Zhejiang Chinese Medical University (Zhejiang Provincial Hospital of Chinese Medicine), Hangzhou, Zhejiang, China; ^2^Department of Intensive Care Unit, The First Affiliated Hospital of Zhejiang Chinese Medical University (Zhejiang Provincial Hospital of Chinese Medicine), Hangzhou, Zhejiang Province, China

**Keywords:** functional dyspepsia, insomnia, auricular acupoint therapy, randomized controlled trial, efficacy

## Abstract

**Background:**

Functional dyspepsia (FD) is a prevalent health issue currently lacking optimal treatment options, with its global incidence rate increasing in recent years. Clinical studies have recently focused on the application of auriculotherapy in functional gastrointestinal disorders that are accompanied by negative emotions. However, few randomized controlled trials have investigated the use of auriculotherapy for FD patients with insomnia, leaving the therapeutic efficacy and safety largely undefined. This study aims to evaluate the clinical efficacy and safety of auriculotherapy in treating FD patients with insomnia.

**Methods and analysis:**

This study is a single-center, randomized controlled clinical trial involving 80 patients with FD and insomnia. Using a central randomization system, the subjects are randomly assigned to the auricular acupressure group or the sham auricular acupressure group at a 1:1 ratio, with the auricular acupressure group targeting the concha region and the sham auricular acupressure group targeting the earlobe region. The primary outcome is the response rate at 2 weeks, and the secondary outcomes include the response rate at 8 weeks, sleep data assessed by actigraphy, modified Functional Dyspepsia Symptom Diary, short form-Nepean Dyspepsia Index, Self-rated Anxiety Scale, Self-rated Depression Scale, High Arousal Scale, and Heart Rate Variability. Efficacy results will be evaluated at baseline and at 2 and 8 weeks after treatment. Adverse events will be monitored throughout the study observation period.

**Discussion:**

The results of this trial are anticipated to validate the efficacy and safety of auriculotherapy in improving symptoms of FD and insomnia, as well as in reducing negative emotional states.

**Clinical trial registration:**

ClinicalTrials.gov, NCT06466044. Registered 14th May 2024, https://register.clinicaltrials.gov.

## 1 Introduction

Functional dyspepsia (FD) is a gastrointestinal disorder characterized by symptoms without identifiable organic changes. According to the Rome IV criteria, FD can be classified into two subtypes: Postprandial Distress Syndrome and Epigastric Pain Syndrome (1, 2). The incidence of FD is significant globally and has been increasing annually (3, 4). Studies indicate that the patterns of FD prevalence vary across different countries and regions, with the incidence rate increasing from 4 to 5% over the past decade (5). Notably, patients in Eastern countries appear to be more susceptible to PDS than compared to their counterparts in Western nations are (6). A research report examining the general populations of Canada, the United States, and the United Kingdom reported an average mixed prevalence rate of 10%, with 61% of these individuals classified as PDS patients. In the United States alone, the economic impact of FD is estimated to exceed 18 billion USD (3). Epidemiological surveys suggest that approximately 30% of individuals with FD are associated with comorbid conditions such as insomnia, particularly among those with a sensitive personality or a genetic predisposition to gastrointestinal motility disorders. Furthermore, many medications used to treat negative mood disorders can themselves induce gastrointestinal side effects (7), which are considered a significant contributor to recurrent symptoms (8–10). Consequently, FD not only severely impairs patients’ quality of life but also imposes a substantial social and economic burden. Thus, the proactive prevention and management of FD, has emerged as a pressing clinical challenge.

Currently, the available treatment options for FDs with insomnia are quite limited in terms of both effectiveness and safety. Commonly utilized treatments for FDs with insomnia focus primarily on enhancing gastrointestinal motility through various agents, while secondary consideration is given to antianxiety and antidepressant medications. Additionally, other approaches include the use of acid-suppressing agents, gastric proteases, and pancreatic enzyme preparations (11, 12). However, the clinical application of these treatments is limited by long-term side effects, ambiguous clinical efficacy, and limited safety across different therapies. These challenges have led many FD patients with insomnia to seek alternative complementary treatments. Although non-pharmacological interventions such as exercise training and music therapy show some promise, their clinical effectiveness and safety require validation through large-scale clinical studies (13). Therefore, the search for clinically effective and safe treatments for FD with insomnia remains an urgent priority.

In recent years, acupuncture treatment has emerged as one of the most prevalent modalities within complementary and alternative medicine (14). Extensive clinical research has been conducted on auricular acupoint therapy (AAT) specifically for functional gastrointestinal disorders associated with negative emotions. AAT is based on the meridian theory and Zang-Fu theory, which are fundamental concepts in traditional Chinese medicine (TCM), and incorporate contemporary medical anatomical knowledge. From the perspective of syndrome differentiation and treatment, specific auricular acupoints are selected. The auricular acupoints are stimulated through moderate rubbing, pressing, pinching, and squeezing of *Vaccaria segetalis* and other medicinal beans adhering to these points, gradually inducing sensations such as soreness, numbness, distension, and pain, to achieve therapeutic objectives. This approach constitutes a type of external treatment in TCM (15). Studies have demonstrated that AAT can relieve constipation and improve anxiety symptoms, thus improving the overall quality of life of patients (16, 17). However, as the application of AAT expands, certain drawbacks have become apparent. For example, most treatment protocols employed in clinical research are based on the personal experiences of researchers, and a standardized protocol has yet to be established. The existing therapies exhibit deficiencies in standardization and reproducibility. Therefore, determining a standardized and unified AAT protocol and exploring the precise efficacy of AAT in treating FD patients with insomnia are crucial.

We conduct a randomized controlled clinical trial at the Zhejiang Provincial Hospital of Chinese Medicine in China. Our objective is to assess the impact of AAT on FD with insomnia. The results of this study will provide valuable insights into the efficacy and safety of AAT for treating FD patients experiencing insomnia.

## 2 Methods and analysis

### 2.1 Study design

**Figure 1:**
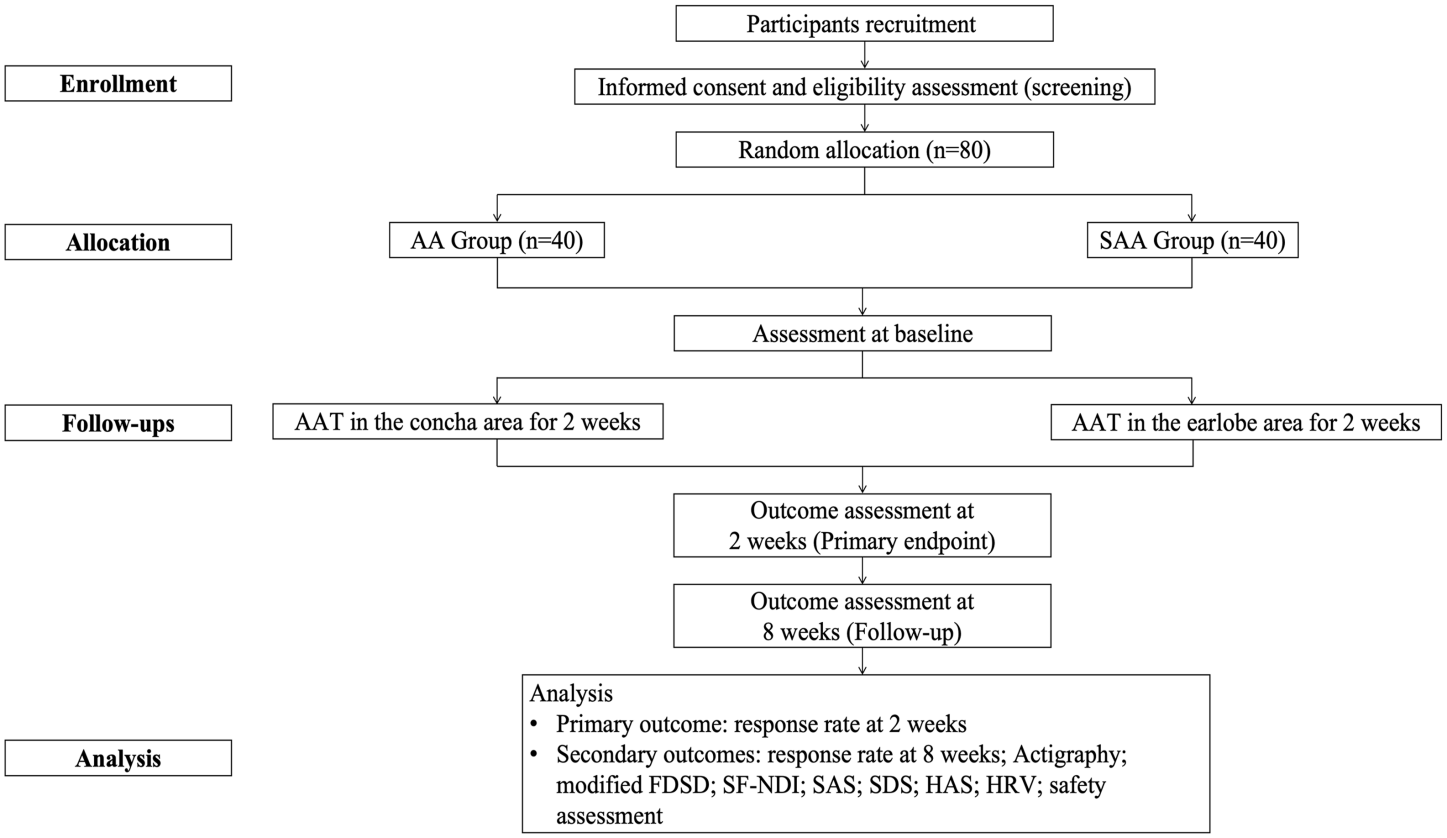
Flowchart of this study.

This study is a single-center, randomized controlled clinical trial designed to evaluate the efficacy and safety of AAT for treating FD patients with insomnia. The trial will take place at the Zhejiang Provincial Hospital of Chinese Medicine. A minimum of 80 eligible patients will be recruited and randomly assigned to one of two groups: one receiving auricular acupressure (AA) targeting the auricular concha area, which has a rich distribution of vagus nerve branches (18), and the other receiving sham auricular acupressure (SAA) in the earlobe area, where the vagus nerve distribution is minimal (19). Following a one-week baseline period, participants will enter a two-week treatment phase, during which they will be observed for up to 8 weeks. The participants will be assessed at three key time points: baseline (1 week prior to treatment), the end of treatment (2 weeks post-treatment), and follow-up (8 weeks post-treatment). Each participant will complete relevant assessments, including the Pittsburgh Sleep Quality Index (PSQI), actigraphy monitoring, modified Functional Dyspepsia Symptom Diary (modified FDSD), short form-Nepean Dyspepsia Index (SF-NDI), Self-Rating Depression Scale (SDS), and the Self-Rating Anxiety Scale (SAS), High Arousal Scale (HAS), Heart Rate Variability (HRV) and safety assessment. Table 1 outlines the schedule for the evaluation, enrollment, and intervention, while Figure 1 presents the flowchart of the study. The study adheres to the SPIRIT reporting guidelines (Supplementary Table 1) (20).

**Table 1:** Trial process chart.

	Baseline	Treatment phase			Follow-up					
	Week	Week	Week	Week	Week	Week	Week	Week	Week	Week
	-1	0	1	2	3	4	5	6	7	8
Patients										
Enrollment	✓									
Signed informed consent	✓									
Baseline feature		✓								
Randomization		✓								
Primary outcome										
Response rate at 2 weeks		✓		✓						
Secondary outcomes										
Response rate at 8 weeks		✓								✓
Actigraphy		✓		✓						✓
modified FDSD		✓		✓						✓
SF-NDI		✓		✓						✓
SAS		✓		✓						✓
SDS		✓		✓						✓
HAS		✓		✓						✓
HRV		✓		✓						✓
Safety		✓	✓	✓	✓	✓	✓	✓	✓	✓

### 2.2 Inclusion criteria

Participants meeting the following criteria will be included:

1. Meets the Rome IV diagnosis criteria for FD (21);
2. Meets the Chinese guideline for diagnosis and treatment of insomnia diagnosis criteria for insomnia (22);
3. PSQI was greater than 7;
4. Aged 18 years or older;
5. Has not taken the following medications for at least 2 weeks before enrollment: antibiotics (oral, intramuscular, and intravenous), probiotics (such as probiotics, prebiotics, and synbiotics) that affect the gastrointestinal microbiota, any medication or health product that improves sleep quality or inhibits brain neural activity, medications related to the treatment of FD or other relevant therapies;
6. The participants agreed to participate in this study and signed an informed consent form.

### 2.3 Exclusion criteria

Participants who reported any of the following conditions will be excluded:

1. Secondary insomnia caused by medication or other diseases;
2. Patients with comorbid mental disorders, as well as severe diseases of the heart, liver, kidneys and other systems;
3. Those who have previously received this treatment method or participated in other clinical trials within the past 6 months;
4. Presence of contraindications for ear acupoint patches, such as skin hypersensitivity or damage to the application site;
5. Pregnant and lactating women.

### 2.4 Recruitment process

Patients will primarily be recruited from the gastroenterology outpatient department of Zhejiang Provincial Hospital of Chinese Medicine. Research assistants will be assigned to assist in the screening of participants. Patients with FD and insomnia who express interest in participating will complete a series of forms under the guidance of a trained physician involved in the trial. Once participants meet the inclusion criteria, patients will be provided with a triaxial accelerometer (GT3X+, wGT3X-BT, ActiGraph, Pensacola, FL) and be instructed to wear the device on their non-dominant wrist for 7 consecutive days to monitor their sleep data, including sleep efficiency (SE), wake time after sleep onset (WASO), sleep onset latency (SL), and total sleep time (TST) using the Cole-Kripke algorithm provided by the manufacturer’s software (Actilife, version 6.11.7). Patients will be provided with instructions to remove the device during periods of strenuous activity and in situations where the device would be in contact with water, including bathing, swimming, and showering. Concurrently, Patients will be asked to maintain a sleep diary to document their in-bed time, out-bed time, and instances when the device was not worn (e.g., during bathing). The diaries are used to cross-reference accelerometer data. Following this, they will be required to sign a written informed consent form before the intervention begins.

### 2.5 HRV and ECG recording

HRV refers to the physiological fluctuations in the time intervals between successive heartbeats. It serves as a crucial indicator of autonomic nervous system function. Participants will be seated in a quiet, well-lit environment and instructed to relax and remain still throughout the recording period to minimize motion artifacts. A resting-state electrocardiogram (ECG) will be recorded continuously for 5 min between 9 AM and 12 noon. The ECG data will be sampled at 1 kHz and filtered online using a main power filter. For each successive heartbeat, the peak R-wave in the ECG will be identified, and the R-R interval will be measured to the nearest millisecond. The Fourier transform method applied to ECG RR intervals will be utilized to calculate both time- domain and frequency-domain indices of HRV.

### 2.6 Sample size calculation

Sample size estimation for the comparison of independent sample rates was conducted via PASS V2023. According to the results of a small sample preliminary experiment, the response rate of the treatment group was 50% (10 cases, 5 of which were effective), and the response rate of the control group was 20% (10 cases, 2 of which were effective). At a significance level of *α* = 0.05 and a power of 1-*β* = 0.8, a minimum of 36 participants was required for each group, assuming equal sample sizes. Considering the expected dropout rate of 10%, the minimum sample size should be 40 participants per group, resulting in a total minimum sample size of 80 participants.

### 2.7 Randomization and blinding

A total of 80 patients diagnosed with FD and insomnia, who meet the inclusion criteria, will be randomly assigned to either the AA or SAA group at a 1:1 ratio via block randomization. A central randomization system will oversee the randomization management process.

Throughout the clinical trials, the subjects, evaluators, data managers, and statisticians will remain unaware of the treatment allocation. Once randomization and allocation are performed by an independent data manager, the information will be conveyed to the professional nurse, who will not participate in evaluating the results or analyzing the data. Neither the study participants nor the investigators influence the randomization process or the concealment of assignments. The participants will be informed of the existence of two treatment options. After 2 months of the study, patients will be asked to complete a blinding assessment, as well as to guess which treatment they received, to evaluate the success of the blinding. All researchers will undergo training on the methodological specifications of this study before the trial and will strictly adhere to the principle of task separation.

### 2.8 Intervention

AAT is a home-based, hospital-supported approach to care, and we will provide approximately 30-min training sessions for participants, including how to perform effective auricular acupressure independently and how to fill out an AA record form. Specifically, after cleaning the skin, a professional nurse will place the cowherb seeds on the ear points and fixes them with tape. The professional nurse will instruct the participants to manually stimulate each acupoint (de qi stimulation) 3 times a day (morning, afternoon and evening), alternately applying to the right and left ear pavilion for 30 s or until the ear pavilion becomes red or sensitive to pressure (23). Each treatment is separated by 1 day. AA record form will be filled out immediately after each acupressure. Considering practical clinical applications, we set 80% of the ideal treatment time (17 sessions) as high adherence. If a patient is in doubt about self-administering treatment at home, help can be obtained by phone or site visit. In addition, patients must receive intensive treatment once a week at the hospital. Each treatment lasts 20 to 25 min in a reserved room. The entire treatment will last 2 weeks. Before patients are enrolled, nurses will be trained in standardization procedures. The entire process will be carried out under the supervision of a professional nurse with at least 10 years of experience in AAT.

#### 2.8.1 Treatment group

**Figure 2:**
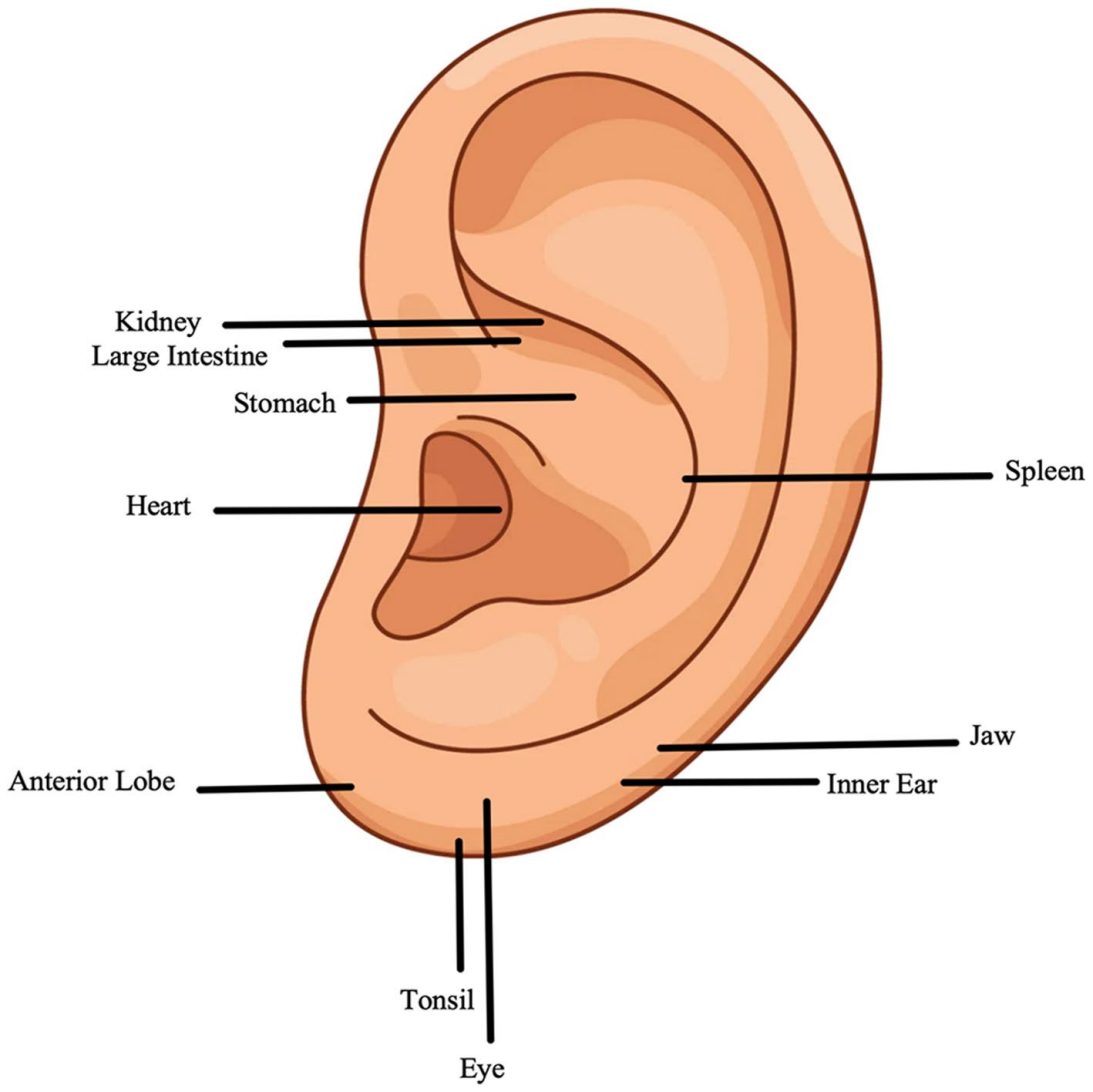
Acupoints selected to treat FD with insomnia.

According to the principles outlined in TCM textbooks, participants in the AA group will receive AAT specifically targeted at the auricular concha area, which is rich in vagus nerve fibers (18). This therapy focuses on the auricular point associated with the seeds of *Vaccaria segetalis*. The auricular acupoints include the Heart (CO15 Xin), Kidney (CO10 Shen), Spleen (CO13 Pi), Stomach (CO4 Wei), and Large Intestine (CO7 Dachang). A detailed illustration of these auricular acupoints can be found in Table 2 and Figure 2.

**Table 2:** Universal nomenclature, location, and indications of the points used in the groups.

Group	Ear acupuncture point / universal and Chinese nomenclature	Anatomical location	Indications
AA	Heart (CO15 Xin)	In the center of the cavum conchae	Used for cardiac complaints such as angina, cardiac rhythm disorders.
	Kidney (CO10 Shen)	In the cymba conchae, in a groove below the start of the inferior crus	Used for issues in the urinary and genital systems. Assists with tinnitus, auditory conditions and sleep disorders.
	Spleen (CO13 Pi)	Located below the BD line, posterosuperior to the concha cavity	Used for digestive system diseases such as abdominal distension and urogenital system diseases such as functional uterine bleeding.
	Stomach (CO4 Wei)	Located at the disappearance of the helix crus	Used for gastritis and other diseases of the digestive system and forehead pain, toothache, insomnia, etc.
	Large Intestine (CO7 Dachang)	In the boat of the concha, medial 1/3 of the upper margin of the helix crus	Used for digestive system diseases such as enteritis and some pulmonary diseases.
SAA	Tonsil (LO8 Biantaoti)	Just below the frontal lobe	Used for tonsillitis, pharyngitis.
	Jaw (LO3 He)	In the positive upside after ear lobes	Used for five sensory disorders such as toothache and temporomandibular joint dysfunction.
	Anterior lobe (LO4 Chuiqian)	In the middle of the earlobe	Used for neurasthenia, toothache and other diseases.
	Eye (LO5 Yan)	In the central part of the earlobe	Used for acute conjunctivitis, electric ophthalmia, stye and other eye diseases.
	Inner ear (LO6 Neier)	In the anteroposterior middle of the earlobe	Used for the ErXing ear diseases such as vertigo, tinnitus, hearing loss.

#### 2.8.2 Control group

Compared with those in the AA group, participants in the SAA group will receive AAT at different locations. Participants in the SAA group will receive AAT specifically targeted at the earlobe area, which is recognized as having the least vagal innervation from the auricular branch (19). The acupoints in the auricular earlobe area include the Tonsil (LO8 Biantaoti), Jaw (LO3 He), Anterior Lobe (LO4 Chuiqian), Eye (LO5 Yan), and Inner Ear (LO6 Neier). These acupuncture points have not been documented in the literature as being able to relieve symptoms of FD or insomnia. These auricular acupoints are listed in Table 2 and Figure 2.

### 2.9 Outcome measurement

#### 2.9.1 Primary outcome

The primary outcome is the response rate based on the proportion of patients with ≥50% reduction in PSQI after 2 weeks of treatment compared to baseline (24). The 2-week outcome could reflect rapid symptomatic relief. The PSQI consists of 19 items, each scored on a Likert scale ranging from 0 to 3. The cumulative score, which can range from 0 to 21, indicates sleep quality, with higher scores reflecting poorer sleep quality and more severe sleep disturbances (25).

#### 2.9.2 Secondary outcomes

1. The response rate at 8 weeks will be assessed via the results from the proportion of patients with ≥50% reduction in PSQI after 8 weeks of treatment compared to baseline. The durability of the treatment effect is evaluated by observation at 8 weeks compared to 2 weeks.
2. Objective sleep state will be conducted at three time points: pre-treatment, 2 weeks post-treatment, and 8 weeks post-treatment. The objective evaluation of sleep patterns was mainly through the use of actigraphy. The actigraphy data will be collected included SE, TST, WASO, and SL. Those data will be processed and analyzed using ActiLife6 V.6.8.1 software (ActiGraph, LLC) to evaluate the patients’ sleep quality (26). SE of less than 85% is considered as poor sleep.
3. The severity of dyspepsia symptoms will be evaluated at three time points: before treatment, 2 weeks after treatment, and 8 weeks after treatment. The total and individual dyspepsia symptom scores will be assessed via the modified FDSD, which comprises five main items: stomach pain, burning in the stomach, bloating, early satiety, and postprandial fullness. Each item can be scored from 0 (no) to 10 (worst) (27).
4. The average change in the SF-NDI will be evaluated at three time points: before treatment, 2 weeks after treatment, and 8 weeks after treatment. The SF-NDI evaluates anxiety (2 items), impact on daily life (2 items), dietary habits (2 items), cognition/self-control (2 items), and work/study (2 items) across five dimensions. Each item is rated on a 5-point Likert scale (1 to 5 points), with a total maximum score of 50. In this context, a higher score denotes more severe symptoms, while a lower score indicates milder symptoms (28).
5. Psychological status assessments will be conducted at three time points: pre-treatment, 2 weeks post-treatment, and 8 weeks post-treatment. The SAS is designed to evaluate somatic symptoms associated with anxiety experienced over the past week, with each item scored on a scale from 1 to 4, including reverse-scored items. A higher total score reflects more severe anxiety symptoms (29). Conversely, the SDS focuses on somatic symptoms related to depression within the same timeframe, employing the same scoring criteria as the SAS. The SDS establishes a cutoff value of 53 points, where scores ranging from 53 to 62 indicate mild depression, scores ranging from 63 to 72 signify moderate depression, and scores exceeding 72 denote severe depression (30).
6. The assessment of autonomic nerve function will be conducted at three time points: pre-treatment, 2 weeks post-treatment, and 8 weeks post-treatment. The effects of AAT on the autonomic nervous function in individuals with FD and insomnia are evaluated via the HAS (31). The HAS consists of 26 items; each item is scored on a scale of 0–3, with higher scores indicating higher levels of cortical arousal. The total score above 32 indicates a state of hyperarousal. HRV will be used to assess the level of autonomic nervous system activity in individuals. The time-domain indicators (SDNN, PNN50) and frequency domain indicators (HF, LF) were collected by HRV. Among them, HF is closely related to vagus nerve activity, and higher HF power values indicate better parasympathetic function and better cardiac vagal tone (32).
7. Potential adverse reactions include allergic reactions to the components of the auriculus patch and localized skin symptoms such as redness, swelling, warmth, pain, itching, and rash. Improper application may result in discomfort, including pain and abrasions. The severity of adverse events is categorized according to the nature of the symptoms: mild (minor apparent discomfort), moderate (adverse events that occasionally interfere with daily activities), and severe (adverse events that persistently interfere with daily activities). All serious adverse events occurring during the study will be reported to the investigator and the ethics committee within 24 h of learning of the event. The research team will take appropriate measures to ensure the safety of participants who experience adverse events, including suspending participants from the study and providing immediate medical care until the adverse event is resolved. In addition, given the potential variability in patient self-management of AAT, in addition to standardized training, each participant will be asked to complete a standardized adverse event reporting form to document adverse events experienced during the study and their severity. We will also conduct regular weekly follow-up visits with all participants. Follow-up will be conducted by telephone interviews and clinic visits to ensure timely identification and documentation. All adverse events will be recorded in detail on a Case Report Form (CRF).

### 2.10 Data management and quality control

Before the study commenced, the Clinical Evaluation Center at this research institution established eCRF within the Electronic Data Capture system. Researchers meticulously input data from the original observation records of subjects into the eCRF to ensure completeness and clarity. Prior to data entry, data managers familiarize themselves with the content and coding of each item in the observation form, meticulously documenting the coding process in a coding log. Database naming conventions are standardized to ensure that they are easily readable and locatable, while also maintaining correctness, security, and confidentiality. Data managers collaborate with principal investigators to develop data range checks and logic checks on the basis of the values of various indicators in the eCRF. They also create corresponding computer programs to prevent erroneous data input prior to entry, identify the reasons for errors, make necessary corrections, and maintain comprehensive records of all errors and modifications. Researchers are prohibited from modifying or accessing data until all participants have been enrolled, observed, and data collected. Finally, assessments are conducted by evaluators who are blinded to group assignments.

Prior to the commencement of the study, all staff underwent specialized training encompassing research objectives, research methodologies, treatment strategies, and quality control protocols. All research documents, including CRFs, treatment records, and sleep diaries, as well as treatment materials, such as *Vaccaria segetalis* seeds, are securely stored at the research site with restricted access. The chief researcher will conduct regular meetings every 3 months to address and resolve any issues encountered during the observation period.

### 2.11 Statistical analysis

All the data in this study will be processed and analyzed via SPSS version 27.0 (SPSS Inc., Chicago, IL, United States) and R project (R, version 4.4.2). All statistical analyses were based on the intentionto-treat population of all randomly assigned patients. The normality of the metric data will be assessed via the Shapiro–Wilk test. If the data exhibit a normal distribution, they will be described using the mean and standard deviation (Mean ± SD); otherwise, the median and interquartile range [M (P25 ~ P75)] will be utilized. Categorical data were summarized using frequencies (percentages). For demographic information, pre- and post-treatment clinical scores, HRV data and sleep data monitored by ActiGraph, if the data follow a normal distribution and demonstrate homogeneity of variance, between-group comparisons were conducted using independent sample t-tests or repeated measures ANOVA, while within-group comparisons will be performed via paired t-tests. In cases where normality or homogeneity is not met, between-group comparisons were conducted using the Mann–Whitney U test, and within-group comparisons utilize the Wilcoxon signed-rank test. Categorical data will be analyzed using chi-square tests for between-group comparisons and paired chi-square tests for within-group comparisons. Ordinal data comparisons will employ the Mann–Whitney U test for between-group analyses and the Wilcoxon signed-rank test for within-group analyses. For missing data, we used multiple imputation under the assumption that the data were missing at random. Repeated measures ANOVA will be used to evaluate differences within the same group across three time points: baseline, the end of 2 weeks of intervention, and the end of the eight-week follow-up. A comprehensive sensitivity analysis, including imputation of missing data and the comparison of different analysis sets (Per-protocol, Modified Intention-to-Treat, and Complete case) of the main outcome, will be conducted to ensure rigorous data analysis and enhance the reliability of our study outcomes. This analysis will be a critical component of our study design aimed at addressing potential data gaps and strengthening the robustness of our findings. Bonferroni correction will be applied to adjust the significance level for multiple comparisons. The Fisher precision test will be employed to assess the success rate of the blind method. All statistical tests will be two-sided, with a significance level set at *p* < 0.05, indicating statistical significance.

## 3 Discussion

Previous randomized controlled trials have consistently focused on acupuncture for the treatment of FD or insomnia, often neglecting the relationship between these two conditions (33, 34). Furthermore, there is a lack of comparable randomized controlled trials investigating AAT for patients with FD with insomnia. Therefore, our study aims to propose a rigorously designed trial to evaluate the efficacy and safety of AAT in this patient population, thereby providing reliable evidence for its use.

According to the Rome IV criteria, functional gastroenteropathy, also referred to as gut-brain interaction disorder, is characterized by physiological dysfunction of the gastrointestinal tract due to the interplay of psychosocial factors via the ‘Gut-Brain Axis’ (35). The Gut-Brain Axis denotes the bidirectional communication network between the brain and the gut, facilitated by the nervous, immune, and hormonal systems. This intricate communication ensures the effective transmission of information between the brain and the gut, which together regulate emotional responses, metabolism, immune function, brain development, and overall brain health, as well as the occurrence of brain diseases (36, 37). Currently, the treatment of FD with insomnia in Western medicine is limited. Medications such as proton pump inhibitors may exacerbate the imbalance of intestinal flora, long-term use may cause many side effects such as dependence, gastrointestinal disorders, and so on. Tricyclic antidepressants and selective serotonin reuptake inhibitors are often associated with dry mouth, constipation, and weight gain (38, 39). While some nonpharmacologic therapies, such as cognitive-behavioral therapy and mindfulness-based stress reduction therapy, have shown promise in addressing the gut-brain axis dysfunction underlying FD and insomnia, they require specialized training, significant time commitment, and high patient compliance (40, 41). In addition, although traditional acupuncture has demonstrated efficacy compared to other alternative therapies, its invasiveness and need for professional management limit widespread adoption, and there is currently a lack of high-quality clinical evidence (42, 43). Therefore, this study, based on the platform of the Gastroenterology Outpatient Department of Zhejiang Hospital of Traditional Chinese Medicine, design a high-quality, single-center, randomized controlled trial, strictly follow the scientific and rigorous experimental design methods, and evaluate the accurate efficacy of AAT for FD accompanied by insomnia based on the clinical scale with high reliability and validity and objective evaluation tools. Thus, the evidence level of the application of AAT in the clinical treatment of FD with insomnia can be improved.

AAT, has garnered increasing attention in recent years. Unlike traditional acupuncture, which employs disposable stainless steel needles to penetrate “acupoints” on the skin and may lead to some bodily harm, AAT offers a less invasive alternative. It has been extensively utilized in the treatment of gastrointestinal diseases. Research has demonstrated that AAT can help relieve gastrointestinal symptoms such as nausea and vomiting and regulate mood (44, 45). Compared with other existing treatments for FD and insomnia, AAT has significant advantages. First, the application of AAT is convenient, easy to handle, has few side effects and low price. In particular, compared with Western medicine treatment, AAT can well overcome the dependence on sites, instruments and equipment when treating FD with insomnia by Western medicine alone, help to improve the accessibility of patients in resource-limited environments, and avoid the side effects caused by long-term medication of patients, increase patient compliance and clinical efficacy, and is suitable for patients seeking safer and long-term treatment. Second, AAT can achieve multi-target adjustment. AAT can improve gastrointestinal function and sleep quality by modulating the neuroendocrine system, which is particularly cost-effective in this disease prone to other comorbidities. Finally, AAT utilizes similar mechanisms as Transcutaneous Auricular Vagus Nerve Stimulation, such as vagus regulation, to provide similar benefits in a non-invasive, patient-friendly manner compared to other alternative therapies. In this study, we administered AAT to the AA group at the Heart, Kidney, Spleen, Stomach, and Large Intestine points. These points are strategically located within the auricular concha region, which is recognized as having the highest concentration of the vagus nerve distribution and has also been shown to be associated with digestive or sleep function. According to TCM, point CO15, Xin, is situated in the central depression of the concha cavity and is believed to nourish blood, promote pulse generation, benefit the heart, and calm the nerves. Similarly, point CO10, Shen, is located in the concha boat, beneath the bifurcation of the upper and lower feet of the helix, and is thought to have analogous effects of nourishing blood, promoting pulse generation, benefiting the heart, and calming the nerves. CO13 Pi is located in the posterior and superior sections of the concha cavity, where it serves to invigorate qi, support the right, regulate the stomach, and facilitate the flow of collaterals. CO4 Wei is found at the vanishing point at the base of the ear, and it functions to promote qi circulation, alleviate food stagnation, clear heat and detoxify, nourish blood, and calm the nerves. CO7 Dachang is situated within the auricles and is effective in clearing heat, purifying the fu-organs, relieving constipation, and preventing diarrhea (16, 45). Numerous studies have confirmed that these acupoints exhibit significant therapeutic effects on FD and sleep disorders (46, 47). For the SAA group, we chose to conduct AAT in the regions of the cheek, jaw, anterior lobe, eye, and inner ear. This approach is evenly distributed across the earlobe region, which is characterized by a sparse distribution of the vagus nerve. These points are intended to access other nerve segments and are not directly associated with symptoms of FD or insomnia. By comparing the outcomes of the SAA and AA groups, we were able to evaluate the true impact of AAT on various parameters such as digestion, sleep quality, and negative mood, while effectively controlling for the influence of the placebo effect. The data from this study will illustrate that AAT in the ear region, where there is a dense distribution of the vagus nerve, has a superior clinical effect (or at least a more favorable trend) compared to AAT in areas with less vagus nerve distribution. Should this study fail to demonstrate statistical significance, we will remain open to the possibility that the findings do not support the original hypothesis. Negative results will be thoroughly analyzed and interpreted through careful examination of the study design, sample size, and the absence of a treatment effect. These results are valuable and necessary, and they do not reflect negatively on the therapy or research designers. Additionally, the statistical significance of proximity warrants further investigation.

In addition, the measurement period could also be seen as a limitation: First, while our study employed a rigorous design to minimize variability, broader clinical application requires consensus on optimal acupoint selection and stimulation parameters. In addition, the specific mechanism of AAT is not fully understood and needs to be further explored through animal studies, sequencing, and neuroimaging. Finally, current data on the long-term efficacy and safety of AAT are limited, and more large-scale, multi-center, long-term studies are needed to verify this.

## 4 Conclusion

The outcomes of this study are anticipated to reveal the clinical effectiveness and safety of AAT in alleviating FD with insomnia. We anticipate that these results can further endorse the use of AAT in the treatment of FD patients with insomnia, thereby providing a scientific theoretical foundation for the clinical application of AAT, and offering reliable evidence and practical recommendations for clinical practice in future research. Simultaneously, it can also promote and implement green diagnosis and treatment approaches, bringing the gospel to more patients and generating significant economic and social benefits.
